# Supplementary material for: What happened and what proves you wrong? Combatting confirmation bias in police investigations through evidence reconstruction and falsification
Source: PLoS One. 2026 Jan 14;21(1):e0327036. doi: 10.1371/journal.pone.0327036 (PMC12820782; doi:10.1371/journal.pone.0327036)
Supplement: S1 Appendix — Instructions and coding scheme for evaluating the open-text responses to the focus manipulation check, the strategy manipulation check, and the suggested next investigative steps. (DOCX) [file pone.0327036.s001.docx]

**Appendix A**

**Coding Scheme**

**Instructions and Coding Scheme for Evaluating the Open-Text Responses to the *Focus* Manipulation Check, the *Strategy* Manipulation Check 2 and the Suggested Next Investigative Steps**

*Dear coder,*

*Your task is to* ***code each participants’ responses to two manipulation checks (the Focus Manipulation Check*** *– hereafter referred to as Manipulation Check 1 (MC1) –* ***and the Strategy Manipulation Check –*** *hereafter referred to as Manipulation Check 2 (MC2))* ***and three next investigative steps they propose*** *should be taken next in a criminal investigation.

In the study, participants read a criminal case. The information is split in two parts: In the beginning, the participants receive initial case information and answer Manipulation Check 1. Then, they receive ambiguous new information about the case and answer Manipulation Check 2. After responding to Manipulation Check 2, they suggest three steps that should be taken next in the investigation.*

***Please read*** *the* ***initial case information*** *and* ***ambiguous new information*** *below carefully, as they provide you with* ***important context*** *information, which you may need for understanding the responses you will code.

Details on how you should take the context information into account are provided alongside the specific coding criteria.*

**Initial Case Information** *(relevant context information for all responses)*

Nina (9 years old) and her friend Mark (12 years old) were playing in the Mariepark in Utrecht. Mark later told the police that the two were grabbed by the neck by an unknown man and dragged about 90 meters into nearby bushes.
In the days following the attack, Mark gave the investigators detailed descriptions of the perpetrator, including information that the man had a noticeably “pimply” face.
Mark said the man tried to rape the children but failed. He then stabbed Mark several times and tried to strangle both children with Mark's shoelaces. Nina suffocated, but Mark survived by playing dead.
Mark left the bushes and sought help from a passer-by. As the passer-by did not have a cell phone himself, he stopped another man, Robert, who then called the police on his cell phone. The call was received at the police station at 17:34.
Mark stated that he saw a brown dog running past the bushes while he was playing dead. After the offender disappeared, Mark managed to crawl out of the bushes.
Mark also reported that shortly before the attack, Nina and he had asked a passer-by what time it was and then made their way to their bikes to ride home. The man said it was a quarter past five.

Robert, the man who alerted the police on the day of the murder, was walking near the crime scene. A few weeks before the incident, it was reported that Robert had offered money to another boy in the same park in exchange for sexual acts. Robert confessed that he had committed the murder and that he had entered the park that day with the intention of seeking sexual contact with children. He later retracted the confession. Investigators argue that many suspects retract their confessions after consulting with their attorney. His records show that he is in therapy for recurring paedophilic thoughts.

Mark, the boy who survived, was of above-average intelligence for his age and it struck investigators as odd that he didn't scream throughout the attack, despite many people walking past the bushes. Investigators argue that it is possible that Mark killed Nina, injured himself and then made the knife disappear. A child protection expert who accompanied Mark during the interrogations told investigators that Mark had “a big secret” - without specifying what that secret was or how he knew. He concluded that Mark's perception of the incident was so clouded due to the highly emotionally charged nature of the situation that his statements, including the description of the perpetrator, could not be relied upon.

**Ambiguous New Information** *(relevant context information for responses from Manipulation Check 2 onwards)*

A new witness has come forward who was walking his brown dog in the park between 5:00 and 6:00 pm on the day of the murder. When the witness was shown a picture of Robert's bike, he said it resembled a bike he had seen near the bushes that day. When Mark was shown a picture of the dog, he confirmed that this was the dog he had seen through the bushes.
Robert works at a warehouse 11 minutes away from the park. Based on the time he clocked out on the day of the murder, the earliest he could have been at the park was 5:27 pm. The day after the murder, Robert was seen by several witnesses near the crime scene. Robert did not fully match the description of the perpetrator given by Mark, and Robert's confession contradicted some of the details in Mark's description of the crime. Robert also stated that the confession was coerced.
Several witnesses stated that they saw a man with a beer can in his hand on a bench near the bushes at the time of the crime. The knife has not yet been found.

**How to provide your scores:**

Please write your scores in the provided Excel file. In the Excel file, all columns that you need to fill in are highlighted in blue. I also added grey columns to the right of these relevant columns, so that you can put an X or something if an answer is confusing/ leave a comment if you don’t know how to score it, then we can look at it together later.


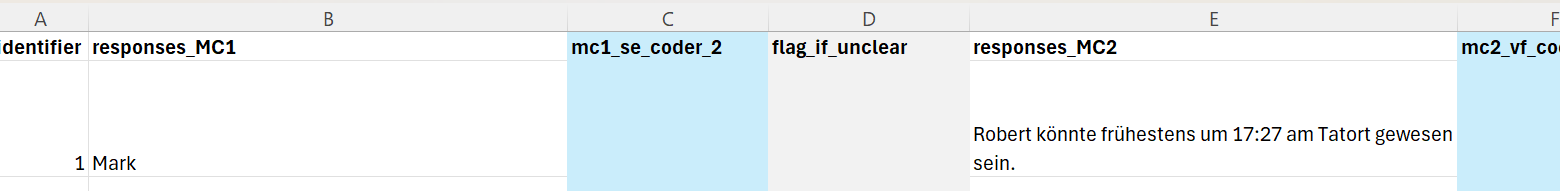


**Coding Criteria MC 1 (Focus)**

*Please evaluate whether each response to Manipulation Check 1 is rather focused on suspects or evidence, using the scale and criteria outlined below.* *When coding the responses to Manipulation Check 1,* ***please keep the initial case information in mind*** *(e.g. when judging if a response is based on the facts from the case file, refer to the initial case information to check if the person stuck to the facts or speculated (a lot)).*

| **Fully Suspect-Focused**  **(-2)** | **Mostly Suspect-Focused**  **(-1)** | **Mixed**  **(0)** | **Mostly Evidence-Focused**  **(+1)** | **Fully Evidence-Focused**  **(+2)** | **NA** |
| --- | --- | --- | --- | --- | --- |
| Mentions/identifies **one most likely suspect**  Lists **pieces of evidence that incriminate** this suspect | - 1. Mentions/identifies **one most likely suspect** | Response contains a **mixture of suspect- and evidence focus** | 1. Presents/considers **two possible scenarios** for what might have happened that are **mostly based on facts from the case file** | 1. presents/considers **two possible scenarios** for what might have happened 2. uses **only facts from the case file** **to reconstruct** what could have happened **like a movie script** | 1. Response contains **a lot of speculations (3 or more)** or is **likely AI generated** 2. Responses for which the **focus is unclear** 3. Responses that **do not fit within** either of the **other categories** |
| **Examples**  *‘Sounds like it must be Robert. He already confessed and was seen near the park. The new witness said the bike looked like his. Mark probably just got confused about the details.’* Scored (-2) because the reasoning is fully centered on one suspect (Robert). The participant selectively uses case details to confirm Robert’s guilt without considering alternative explanations or reconstructing the event.  **Other examples:**  *I think Robert did it because he has paedophilic interests and confessed to the murder.*  or  *I think an unknown third person did it because Robert retracted his confession, and Mark could likely not have stabbed himself multiple times.* | **Examples**  *Mark did it.*  or  *I think Robert is the most likely perpetrator.* | **Example**   - Response presents two scenarios but also identifies a most likely perpetrator - Response presents two scenarios but in both scenarios, the same person is considered the perpetrator   *‘Maybe Mark hurt Nina by accident while they were playing, then panicked and made up the story about a man in the bushes to cover it up. He could have given himself stab wounds to make the story seem more believable. Or Mark meant to hurt her and got the wounds from Nina when she was fighting him in self-defense.’* Scored ‘0’ because while the response presents two scenarios in both scenarios, the same person (Mark) is considered the perpetrator. | **Examples**   - Responses that present two scenarios but don’t reconstruct what happened like a movie script, e.g.   *Scenario 1 – Robert killed Nina and stabbed Mark.*  *Scenario 2 – Mark was the perpetrator and tried to cover up his crime.*   - Scenarios that are written like a movie script but contain some (1-2) speculations about motives or background of the involved persons, e.g. *Mark was psychotic and killed Nina* | **Example**  *‘Scenario 1: Robert approached the children near the path, tried to drag them into the bushes, then fled after the attack Scenario 2: A third person (maybe guy who had been drinking near the bench?) grabbed the children and ran into the bushes while Robert was still elsewhere in the park’* Scored ‘+2’ because two possible scenarios for what might have happened are considered, using only facts from the case file to reconstruct what could have happened as a sequence of events - similar to a movie script  **Other examples:**  *Scenario 1 – Robert left work and biked straight to the park. There, he dragged the kids into the bushes, tried to rape them, but failed. He killed Nina, but Mark escaped and sought help.*  *Scenario 2 – Mark dragged Nina into the bushes and killed her. He hurt himself to make it seem like he was a victim and went to seek ‘help’ from a passer-by.* | **Examples**   - Response presents two scenarios that are far removed from the facts from the case file and based on strong speculations (e.g. ‘*Mark had enough of Nina and wanted to get rid of her*’) - Responses that are likely AI-generated (indicators can be: very extensive, extremely well structured, formatting that’s typical for e.g. ChatGPT) - Response identifies one most likely suspect but also expresses serious doubts about that choice - response clearly identifies one most likely suspect but lists evidence for and against this person |

**Coding Criteria MC 2 (Strategy)**

*Please evaluate whether each response to Manipulation Check 2 verifies or falsifies the response from Manipulation Check 1, using the scale and criteria outlined below.*

*To make this evaluation,* ***consider the participant’s response to Manipulation Check 1****. For example: If a participant states in Manipulation Check 1, ‘I think Mark did it’, and in Manipulation Check 2, ‘Robert couldn’t have made it to the park in time’, the Manipulation Check 2 response is verification-focused, as it lists a point that reinforces the assumption that Mark could be the perpetrator by ruling out the alternative suspect, Robert. Conversely, if the participant’s Manipulation Check 1 response was ‘Robert did it’, then ‘Robert couldn’t have made it to the park in time’ would be falsification-focused, as it lists a point that contradicts the initial assumption.*

| **Fully Verification-Focused**  **(-2)** | **Partially Verification-Focused**  **(-1)** | **Mixed**  **(0)** | **Partially Falsification-Focused**  **(+1)** | **Fully Falsification-Focused**  **(+2)** | **NA** |
| --- | --- | --- | --- | --- | --- |
| Response **lists concrete points that support previous assumptions** from MC 1 or concretely explains how the new evidence supports the previous response.  **Examples**  [*I think Robert is the perpetrator*] *– His bike was seen near the crime scene, and he worked nearby so he could have made it to the park in time to commit the crime*  [*Scen. 1: Robert did it* *Scen. 2: Mark did it*] – *What supports my first scenario is … What supports my second scenario is ...*  Information in square brackets […] might have to be taken from MC1. | Response **states that assumptions from MC 1 are supported** but **doesn’t mention specific points** that support the assumptions.  or  responses **that most likely** (but not certainly) **verify** the prior response.  **Examples**   - *The new information increases/confirms my suspicion that Mark did it* - *Response MC 1: Mark did it* *Response MC 2: Robert couldn’t have done it because he wasn’t at the park in time* (likely, but not explicitly verifies the assumption that Mark is the perpetrator) | response contains a **mixture of verification- and falsification focus.**  **Examples**   - lists some ***points that support*** the initial assumptions/ reconstruction, but ***also*** mentions ***points that contradict*** them - says that the new information ***supports*** one scenario but ***contradicts*** the other | Response **states that assumptions from MC 1 are contradicted or expresses uncertainty** about them but **doesn’t mention concrete points** that contradict these assumptions.  or  responses that **most likely** (but not certainly) **falsify** the prior response.  **Examples**   - *The new information makes it seem less likely that…/* *The new information contradicts my reconstructions.* | Response **lists concrete points that contradict previous assumptions** from MC 1 or concretely explains how the new evidence contradicts the previous response.  **Examples**  [*I think Robert is the perpetrator*] *– Mark’s description of the perpetrator doesn’t match Robert, and the time window makes it unlikely that he could have made it to the park in time*  [*Scen. 1: Robert did it* *Scen. 2: Mark did it*] – *What contradicts my first scenario is … What contradicts my second scenario is ...*  Information in square brackets […] might have to be taken from MC 1. | It **doesn’t become clear** – based on the response to MC 2 and the context provided by MC 1 – **if the response verifies or falsifies** a prior assumption/reconstruction  or  **response doesn’t fit within either of the other categories.**  **Examples**   - MC 1 presents two scenarios: *1) Mark did it, 2) Robert did it.* The response to MC 2 is ‘*time of crime, confession*’ – here it wouldn’t be clear if these points aim to verify or falsify if Robert or Mark could have done it. - **two scenarios** are **reconstructed** in MC 1, **but only one** of them is **mentioned in the response to MC 2**   says that the new information ***contradicts*** one scenario but ***doesn’t contradict*** the other. |

**Coding of Next Investigative Steps**

*Each participant suggested three next investigative steps.* ***Please assign two separate scores to each step****:*

*1)* ***Suspect- vs. Evidence-Focus:*** *Is the step focused on suspects- or evidence?*

*2)* ***Verification- vs. Falsification-Focus:*** *Is the step aimed at verifying or falsifying prior assumptions or (aspects of) reconstructions?*

**General approach:**

1. **Read the suggested investigative step carefully**:
   - Each step consists of **two parts**: *what* should be done and *why*. These are found in separate columns in the Excel file (*stepX_what* & *stepX_why*)
   - Always consider both parts together when assigning the scores
2. **Rate the suspect vs. evidence focus of the step** using the criteria outlined below
3. **Assign a verification vs. falsification score for the same step** using the criteria outlined below
4. Move on to the next step

**Important Considerations**:

- - **Sometimes, the focus/foci of the step become(s) immediately clear when reading the step**

Example: *‘Investigate Robert’s phone – he is the main suspect, and his phone may contain incriminating evidence.’*

- - - Here, the assumption clearly is that Robert is the perpetrator (*suspect-focus*), and the step clearly aims to verify this assumption by seeking evidence that incriminates Robert (*verification-focus*)
    - **If the focus is clear based on the step alone** (e.g. because the underlying assumptions are obviously stated) **you can assign the scores without additional context**
  - **If the focus is not immediately clear, go back to the participant’s responses to Manipulation Check 1 and Manipulation Check 2 for additional context**

Example: *‘Talk to Robert again.’*

- - - Without context, it is unclear whether Robert is considered a suspect (suspect-focus) or a witness who might provide new evidence (evidence-focus)
    - In cases like this, check the participant’s responses to Manipulation Check 1 and Manipulation Check 2 to see if the provided context clarifies the participant’s perspective on the case
  - **If the underlying assumption remains unclear even after checking Manipulation Check 1 and Manipulation Check 2, code the step as *NA***
    - E.g., if the step said ‘*Talk to Robert again*’ but the responses to Manipulation Check 1 and Manipulation Check 2 also do not specify if Robert is seen as a suspect or a witness
    - See grading criteria for more examples and further details on how to handle such cases

**Coding Criteria: Suspect- vs. Evidence-Focus of Suggested Next Steps**

| **Fully Suspect-Focused**  **(-2)** | **Mostly Suspect-Focused**  **(-1)** | **Mixed**  **(0)** | **Mostly Evidence-Focused**  **(+1)** | **Fully Evidence-Focused**  **(+2)** | **NA** |
| --- | --- | --- | --- | --- | --- |
| 1. The step **clearly focuses on/centres around investigating a particular suspect** (whose guilt is assumed). | The primary aim of the step is to **generate new insights/**evidence **that are/**is **directly linked to one suspect or their guilt.** | The step contains a **mixture of suspect- and evidence focus.** | The primary aim of the step is to **generate new evidence,** which may **secondarily be used to gain insights into** **different suspect’s** **or** a **general ‘perpetrator’s’** **involvement** in the case. | The step **clearly focuses on generating new evidence that is neutral and not linked to any particular suspect** – for example to provide new perspectives on the case. | It **doesn’t become clear** – based on the step itself or with the help of the context provided by responses to MC 1 and MC 2 – if the step centres around suspects- or evidence  or  the step **does not fit within** either of the **other categories.** |
| **Example**  [Context from MC 1 and MC 2: Robert is considered the most likely perpetrator] – *Search Robert’s home for the knife.* | **Example**  [Context from MC 1 and MC 2: Robert is considered the most likely perpetrator] – *Talk to other people in the park if they saw Robert near the bushes.* | **Example**  *Find the man with the beer can – he could be a* ***witness*** ***or*** *the* ***perpetrator****.* Here, a specific person is investigated who is identified as a potential perpetrator (suspect-focus), but he is also considered a potential witness (which would make talking to him rather evidence-focused). Thus, this step would be coded as mixed. | **Example**  [Insights into different suspect’s involvement]: *Find the knife to see if the fingerprints match Robert or Mark.*  or  [Insights into a general ‘perpetrator’s’ involvement]: *Search for DNA, so that the perpetrator can be identified.* | **Example**  *Check CCTV footage of the area to see if it provides any additional insights.*  or  *Take all kinds of samples from the crime scene and the people in the area to secure all traces.* | **Examples**   - **two steps/actions are suggested** within one step (that have a different focus), e.g., *Search Robert’s house (suspect) and find new witnesses in the park (evidence)* |

**Coding Criteria: Verification- vs. Falsification-Focus of Suggested Next Steps**

| **Fully Verification-Focused**  **(-1)** | **Neutral**  **(0)** | **Fully Falsification-Focused**  **(+1)** | **NA** |
| --- | --- | --- | --- |
| 1. The step **clearly aims to verify or generate information that supports/ is in line with prior assumptions.** (This becomes clear based on the step itself or based on the context provided by MC 1 and MC 2.) | The step **neither** **explicitly** aims to **verify nor falsify** any **assumptions** or (aspects of) the reconstructed scenarios. | The step **clearly aims to falsify or generate information that challenges** (aspects of) **prior assumptions. (**This becomes clear based on the step itself or based on the context provided by MC 1 and MC 2.)  or  The step aims to **generate insights that are used to differentiate** between **different suspects/scenarios**. | It **doesn’t become clear** – based on the step itself or with the help of the context provided by responses to MC 1 and MC 2 – **if the step aims to verify or falsify** a prior assumption or (aspects of) the reconstruction(s)  or  The step **doesn’t fit within either of the other categories** |
| **Examples**  [Clear based on the step itself:]  *Search Robert’s home, he could have the knife.*  [Clear based on the context:] Suggested step: *Have Mark examined by a psychologist to see if he could have done it.*  Context: *Two scenarios considered in MC 1 and MC 2: ‘Mark did it’ vs. ‘Robert did it’.* In this case, it becomes clear based on the context that the step intends to verify the scenario in which Mark is the perpetrator. | **Examples** *Check CCTV footage in the surrounding area to see if there are some new insights.*  *Find the knife – if there are fingerprints on it, then the police can use those to determine the perpetrator/who held it.*  These two steps are completely neutral in the sense that they do not actively try to confirm or challenge any assumptions. They just neutrally state that the insights gained through the step will help advance the investigation in some way. | **Examples**  [Clear based on the step itself:]  *Question Robert’s colleagues if he left work straight after clocking out – if he didn’t, he couldn’t have made it to the park in time.*  [Clear based on the context provided in MC 1 and MC 2:]  Suggested step: *Have Mark examined by a psychologist to see if he could have done it.*  Context: *Robert is considered the most likely perpetrator*  Because the respondent assumes that Robert is the most likely perpetrator, checking if Mark could have done it challenges the suspicion towards Robert.  [Generating information with the explicit purpose of discriminating between scenarios/suspects:] *Find out if Mark’s injuries could have been self-inflicted or not – this could confirm or refute his version of events.*  *Secure DNA from the crime scene. If Robert’s DNA is found there, this would suggest he was involved. If it isn’t, he can be ruled out as a suspect.* | **Examples**  **Steps for which the focus doesn’t become clear, even after looking at the responses to MC 1 and MC 2 for context**, e.g., *Check if Robert could have been at the park in time* (context: Robert is assumed to be the perpetrator). Checking if he could have been at the park in time could confirm his guilt (showing he was there) or disconfirm it (proving he couldn’t have arrived in time), which makes the focus unclear.  **Steps that seek to clarify things, but it does not become clear from the phrasing if the aim is to verify or falsify that aspect of the case** (e.g., ‘*clarify/determine if Robert could have been there in time*’) |
