# Supplementary material for: What happened and what proves you wrong? Combatting confirmation bias in police investigations through evidence reconstruction and falsification
Source: PLoS One. 2026 Jan 14;21(1):e0327036. doi: 10.1371/journal.pone.0327036 (PMC12820782; doi:10.1371/journal.pone.0327036)
Supplement: S2 Appendix — Full case vignette, manipulation texts used across conditions, and demographic questions. (DOCX) [file pone.0327036.s002.docx]

**Appendix B**

**Materials Main Study**

This appendix includes all materials used in the main study. As the study was conducted in German, we first present English translations of the materials to facilitate accessibility for an international audience. The original German versions are provided thereafter.

**Materials – English**

**Initial Case Information**

Please read the following case carefully. Later in the study you will be asked to help investigate the crime. Since this case is based on a real event, names and dates have been modified to protect the identities of the real persons involved.

Nina (9 years old) and her friend Mark (12 years old) were playing in Mariepark in Utrecht (Netherlands). Mark later told the police that the two were grabbed by the neck by an unknown man and dragged about 90 meters into nearby bushes.

In the days following the attack, Mark gave the investigators detailed descriptions of the perpetrator, including the information that the man had a noticeably ‘pimply’ face.

Mark said the man tried to rape the children but failed. He then stabbed Mark several times and tried to strangle both children with Mark's shoelace. Nina suffocated, but Mark survived by playing dead.

Mark left the bushes and sought help from a passer-by. As the passer-by did not have a cell phone himself, he stopped another man, Robert, who then called the police on his cell phone. The call was received at the police station at 17:34.

Mark stated that he saw a brown dog running past the bushes while he was playing dead. After the attacker disappeared, Mark managed to crawl out of the bushes.

Mark also reported that shortly before the attack, Nina and he had asked a passer-by what time it was and then made their way to their bikes to go home. The man said it was quarter past five.

***Suspect 1***

Mark, the boy who survived, was very intelligent for his age and it struck investigators as odd that he did not scream throughout the attack, even though many people walked past the bushes. Investigators argue that it is possible that Mark killed Nina, injured himself and then made the knife disappear. A child protection expert who accompanied Mark during the interrogations told investigators that Mark had ‘a big secret’ – without specifying what that secret was or how he knew. He concluded that, due to the highly emotionally charged nature of the situation, Mark's perception of the incident was so blurred that his statements, including the description of the perpetrator, could not be relied upon.

***Suspect 2***

Robert, the man who alerted the police on the day of the murder, was walking near crime scene. A few weeks before the incident, it was reported that Robert had offered money to another boy in the same park in exchange for sexual acts. Robert confessed that he had committed the murder and that he had entered the park that day with the intention of seeking sexual contact with children. He later retracted the confession. Investigators argue that many suspects retract their confessions after consulting with their lawyer. Robert’s files show that he is in therapy for recurring paedophilic thoughts.

**Manipulation Part 1 [suspect- vs. evidence-focus]**

***Who did it? [Suspect-Focused]***

Instructions (Read carefully):
**Your task is to find out who committed the crime.** Look carefully at all the available evidence and pay attention to any clues that might point to the real perpetrator. **Decide on ONE person you think is the most likely perpetrator and list evidence that could be used to incriminate that person.**

If you want to freshen up your memory of the case, you will find the case information below the text field.

***What could have happened? [Evidence-Focused]***

Instructions (Read carefully):
**Develop two possible scenarios for what could have happened.** **Focus on the facts** from the case file and **avoid speculating too much about possible suspects**. Your aim is to reconstruct two scenarios using the available information. Imagine you are writing a **script for a movie** (1 “script” per scenario). Outline the sequence of events for each of the two scenarios.

If you want to freshen up your memory of the case, you will find the case information below the text field.

**Ambiguous New Evidence**

**There is new information regarding the case!** Please read the information carefully.

A new witness has come forward who was walking his brown dog in the park between 17:00 and 18:00 on the day of the murder. When the witness was shown a picture of Robert's bike, he said it resembled a bike he had seen near the bushes that day. When Mark was shown a picture of the dog, he confirmed that this was the dog he had seen through the bushes.

Robert works at a warehouse 11 minutes away from the park. Based on the time he clocked out on the day of the murder, the earliest he could have been at the park was 5:27 pm. The day after the murder, Robert was seen by several witnesses near the crime scene. Robert did not fully match the description of the perpetrator given by Mark, and Robert's confession contradicted some of the details in Mark's description of the crime. Robert also stated that the confession was coerced.

Several witnesses stated that they saw a man with a beer can in his hand on a bench near the bushes at the time of the crime. The knife has not yet been found

**Manipulation Part 2 [verification- vs. falsification-focus]**

***What proves you right? [Verification-Focused]***

***[Verification- x Suspect-Focus]***

Please list all points from the new case information that **support** **your** initial **assessment of the perpetrator**.

***[Verification- x Evidence-Focus]***

Please list how the new case information **supports** **the different scenarios** you have developed for the course of the crime.

***What proves you wrong? [Falsification-Focused]***

***[Falsification- x Suspect-Focus]***

Please list all points from the new case information that **contradict your** original **assessment of the perpetrator**.

***[Falsification- x Evidence-Focus]***

Please list how the new case information **contradicts** **the different scenarios** you have developed for the course of the crime.

**Next Investigative Steps**

Now, please suggest **three specific actions or investigative steps** that you think should be taken next to move the investigation forward.

Please **indicate for each step:**

1. **What should be done:** Provide detailed and specific steps that could move the investigation forward
2. **Why is this necessary/how do the steps advance the investigation:** Explain how each step advances the investigation or what insights they provide for the investigation

**Step X: What should be done?**

[Text Box – at least 25 signs]

**Step X: Why?** (How does this move the investigation forward?)

[Text Box – at least 50 signs]

**Guilt Ratings**

***Sliders***

Without knowing the outcome of the next steps you suggested, please indicate how certain you are that each of the following suspects is the perpetrator?

*Robert* – slider from 0 (not guilty) to 100 (certainly guilty)

*Mark* – slider from 0 (not guilty) to 100 (certainly guilty)

*Someone else* – slider from 0 (not guilty) to 100 (certainly guilty)

***Text Box***

Why do you think so? (Please explain your final assessment of guilt).

**Attention Checks**

1. How did the children initially notice the time?
   1. They checked their watches
   2. **They asked a passerby**
   3. They saw it on a nearby clock
   4. It didn’t say
2. Who is Nina?
   1. The person calling the authorities
   2. One of the suspects
   3. **The victim who died**
   4. The mother of one of the children
3. What is the occupation of Robert?
   1. Police officer
   2. Teacher
   3. Painter
   4. **It didn’t say**
4. What was the witness doing in the park?
   1. Talking to his neighbour
   2. Exercising
   3. Having a picnic
   4. **Walking his dog**

**Demographic Questions & Covariates**

1. How old are you?
2. What is your gender?
3. What is your level of police training?
   1. I am still in training
   2. Intermediate Service (*Mittlerer Dienst*)
   3. High Service (*Gehobener Dienst*)
   4. Higher Service (*Höherer Dienst*)
4. How many years of practical experience do you have in police work?

[Input field] _____ years (entries in 0.5 steps are possible)

1. Are you or have you been working fort the Criminal Investigation Office?

☐ Yes ☐ No

**Materials – German**

**Erste Fallinformationen**

**Bitte lesen Sie den folgenden Fall sorgfältig durch. Sie werden später gebeten, bei der Aufklärung des Verbrechens zu helfen.** Der Fall baisert auf einem realen Ereignis. Namen und Daten wurden geändert, um die Identität der beteiligten Personen zu schützen.

Nina (9 Jahre alt) und ihr Freund Mark (12 Jahre alt) spielten im Mariepark in Utrecht. Mark sagte später der Polizei, dass die beiden von einem unbekannten Mann im Nacken gepackt und etwa 90 Meter in ein nahegelegenes Gebüsch gezerrt wurden.
In den Tagen nach dem Angriff gab Mark den Ermittlern detaillierte Beschreibungen des Täters, inklusive der Information, dass der Mann ein auffallend „pickeliges“ Gesicht gehabt hätte.
Mark sagte, der Mann habe versucht die Kinder zu vergewaltigen, scheiterte jedoch. Daraufhin habe er mehrfach auf Mark eingestochen und versucht, beide Kinder mit Marks Schnürsenkel zu erwürgen. Nina erstickte, aber Mark überlebte, indem er sich tot stellte.
Mark verließ das Gebüsch und suchte Hilfe bei einem Passanten. Da der Passant selbst kein Handy besaß, stoppte er einen weiteren Mann, Robert, der daraufhin mit seinem Handy die Polizei anrief. Der Anruf ging um 17:34 in der Polizeiwache ein.
Mark gab an, dass er einen braunen Hund am Gebüsch vorbei laufen sah, während er sich tot stellte. Nachdem der Täter verschwunden war, schaffte es Mark, aus dem Gebüsch zu kriechen.
Mark berichtete auch, dass Nina und er kurz vor dem Angriff einen Passanten nach der Uhrzeit gefragt und sich daraufhin auf den Weg zu ihren Fahrrädern gemacht haben, um nach Hause zu fahren. Der Mann sagte, es sei viertel nach fünf.

***Verdächtiger 1***

Mark, der Junge, der überlebt hat, war für sein Alter überdurchschnittlich intelligent und es kam den Ermittlern seltsam vor, dass er während des gesamten Angriffs nicht geschrien hat, obwohl viele Menschen am Gebüsch vorbeigegangen sind. Die Ermittler argumentieren, dass es möglich ist, dass Mark Nina getötet, sich selbst verletzt und anschließend das Messer verschwinden lassen hat. Ein Kinderschutzexperte, der Mark während der Verhöre begleitete, sagte den Ermittlern, Mark habe „ein großes Geheimnis“ – ohne zu spezifizieren, was dieses Geheimnis sei oder woher er das wisse. Er kam zu dem Schluss, dass Marks Wahrnehmung des Vorfalls aufgrund der hohen emotionalen Belastung der Situation so stark getrübt sei, dass man sich auf seine Aussagen, einschließlich der Täterbeschreibung, nicht verlassen könne.

***Verdächtiger 2***

Robert, der Mann, der am Tag des Mordes im Park die Polizei alarmierte, war in der Nähe des Tatorts unterwegs. Einige Wochen vor dem Vorfall wurde berichtet, dass Robert im selben Park einem anderen Jungen Geld im Gegenzug für sexuelle Handlungen angeboten hatte. Robert gestand, dass er den Mord begangen habe und dass er an diesem Tag den Park in der Absicht betreten hatte, sexuellen Kontakt zu Kindern zu suchen. Später zog er das Geständnis zurück. Die Ermittler argumentieren, dass viele Verdächtige nach Rücksprache mit ihrem Anwalt ihre Geständnisse widerrufen. Seine Akten zeigen, dass er sich wegen wiederkehrender pädophiler Gedanken in Therapie befindet.

**Manipulation Teil 1 [Verdächtigen- vs. Beweis-Fokus]**

***Wer war es? [Verdächtigen-Fokus]***

Bitte lesen Sie die folgenden Anweisungen aufmerksam durch:
**Ihre Aufgabe ist es, herauszufinden, wer das Verbrechen begangen hat.** Betrachten Sie alle vorliegenden Beweise sorgfältig und achten Sie auf alle Hinweise, die auf den wahren Täter hindeuten könnten. **Entscheiden Sie sich für EINE Person, von der Sie denken, dass sie der wahrscheinlichste Täter ist und listen Sie Beweise, mit der man diese Person belasten könnte.**
Sollten Sie nochmal etwas nachlesen wollen, finden Sie die Fallinformationen unter dem Textfeld.

***Was könnte passiert sein? [Beweis-Fokus]***

Bitte lesen Sie die folgenden Anweisungen aufmerksam durch:
Entwickeln Sie **zwei mögliche Szenarien** dafür, **was passiert sein könnte**. **Fokussieren Sie sich auf die Fakten** aus der Fallakte und **vermeiden Sie es**, zu sehr über mögliche Verdächtige **zu spekulieren**. Ihr Ziel ist es, zwei Szenarien mithilfe der vorhandenen Informationen zu rekonstruieren. Stellen Sie sich dabei vor, Sie würden ein **Drehbuch für einen Film** schreiben (1 "Drehbuch" pro Szenario). Stellen Sie dabei die Reihenfolge der Geschehnisse für jedes der beiden Szenarien dar.
Sollten Sie nochmal etwas nachlesen wollen, finden Sie die Fallinformationen unter dem Textfeld.

**Mehrdeutige Neue Informationen**

**Es gibt neue Informationen!** Bitte lesen Sie die Informationen sorgfältig:
Ein neuer Zeuge hat sich gemeldet, der am Tag des Mordes zwischen 17:00 und 18:00 Uhr mit seinem braunen Hund im Park spazieren gegangen ist. Als dem Zeugen ein Bild von Roberts Fahrrad gezeigt wurde, sagte er, es ähnele einem Fahrrad, das er an diesem Tag in der Nähe der Büsche gesehen habe. Als Mark ein Bild des Hundes gezeigt wurde, bestätigte er, dass dies der Hund war, den er durch das Gebüsch gesehen hatte.
Robert arbeitet in einem Lagerhaus, das 11 Minuten vom Park entfernt ist. Basierend auf der Zeit, zu der er am Tag des Mordes ausgestempelt hat, könnte er frühestens um 17:27 Uhr im Park gewesen sein. Am Tag nach dem Mord wurde Robert von mehreren Zeugen in der Nähe des Tatorts gesehen. Robert passt nicht vollständig zu der von Mark gegebenen Täterbeschreibung und Roberts Geständnis widersprach einigen Details aus Mark’s Beschreibung des Tathergangs. Robert gab außerdem an, dass das Geständnis erzwungen worden sei.
Mehrere Zeugen gaben an, zur Tatzeit einen Mann mit einer Bierdose in der Hand auf einer Bank in der Nähe der Büsche gesehen zu haben. Das Messer wurde noch nicht gefunden.

**Manipulation Teil 2 [Verifikations- vs. Falsifikations-Fokus]**

***Was beweist, dass Sie recht haben? [Verifikations-Fokus]***

***[Verifikations- x Verdächtigen-Fokus]***

Bitte listen Sie alle Punkte aus den neuen Fallinformationen, die Ihre **ursprüngliche Einschätzung zum Täter stützen**.

***[Verifikations- x Beweis-Fokus]***

Bitte listen Sie auf, wie die neuen Fallinformationen die **unterschiedlichen Szenarien**, die Sie für den Tathergang entwickelt haben, **stützen**.

***Was zeigt, dass Sie falsch liegen? [Falsifikations-Fokus]***

***[Falsifikations- x Verdächtigen-Fokus]***

Bitte listen Sie alle Punkte aus den neuen Fallinformationen, die Ihrer **ursprüngliche Einschätzung zum Täter widersprechen**.

***[Falsifikations- x Beweis-Fokus]***

Bitte listen Sie auf, wie die neuen Fallinformationen den **unterschiedlichen Szenarien**, die Sie für den Tathergang entwickelt haben, **widersprechen**.

**Nächste Ermittlungsschritte (Next Investigative Steps)**

**Bitte schlagen Sie nun drei konkrete Maßnahmen oder Ermittlungsschritte vor**, die Ihrer Meinung nach als nächstes unternommen werden sollten, um die Untersuchung voranzubringen.
Bitte geben Sie **für jeden Schritt** an:
• **Was sollte gemacht werden**: Geben Sie detaillierte und konkrete Schritte an, die die Untersuchung voranbringen könnten
• **Warum ist das notwendig/wie bringen die Schritte die Untersuchung voran**: Erklären Sie, wie die Schritte die Untersuchung voranbringen, bzw. welche Erkenntnisse sie für die Untersuchung liefern

**Schritt X: Was sollte gemacht werden?**

[Textfeld– mindestens 25 Zeichen]

**Schritt X: Warum?** (Wie bringt das die Untersuchung voran?)

[Textfeld – mindestens 50 Zeichen]

**Schuldeinschätzung**

***Schieberegler***

Ohne zu wissen, welche neuen Informationen die von Ihnen vorgeschlagenen nächsten Schritte geliefert hätten, **geben Sie bitte an, wie sicher Sie zum jetzigen Zeitpunkt sind, dass die folgenden Verdächtigen den Mord begangen haben.**

*Robert* – Schieberegler von 0 (nicht schuldig) bis 100 (sicher schuldig)

*Mark* – Schieberegler von 0 (nicht schuldig) bis 100 (sicher schuldig)

*Someone else* – Schieberegler von 0 (nicht schuldig) bis 100 (sicher schuldig)

***Textfeld***

Warum glauben Sie das? (Bitte erklären Sie ihre finale Schuldeinschätzung)

[Textfeld]

**Aufmerksamkeitschecks**

1. Woher wussten die Kinder, wie spät es ist?
   1. Sie sahen auf ihre Uhren
   2. **Sie fragten einen Passanten**
   3. Sie sahen auf eine nahegelegene Uhr
   4. Das wurde nicht gesagt
2. Wer ist Nina?
   1. Die Person, die die Polizei alarmierte
   2. Eine der Verdächtigen
   3. **Das Opfer, das starb**
   4. Die Mutter eines der Kinder
3. Was ist Robert’s Beruf?
   1. Polizist
   2. Lehrer
   3. Maler
   4. **Das wurde nicht gesagt**
4. Was machte der Zeuge im Park?
   1. Mit seinem Nachbarn sprechen
   2. Sport treiben
   3. Ein Picknick machen
   4. **Mit seinem Hund spazieren gehen**

**Demographische Fragen & Kovariaten**

1. Wie alt sind Sie?

[Eingabefeld] _____ Jahre (Angaben in ganzen Jahren)

1. Mit welchem Geschlecht identifizieren Sie sich am ehesten?
   1. Weiblich
   2. Männlich
   3. Divers
   4. Keine Angabe
2. Was ist Ihr höchster Abschluss innerhalb der Polizei??
   1. Ich befinde mich noch in der Ausbildung
   2. Abgeschlossene Ausbildung zum Mittleren Dienst
   3. Abgeschlossene Ausbildung zum Gehobenen Dienst
   4. Höherer Dienst
   5. Sonstige
3. Wie viele Jahre praktische Berufserfahrung im Polizeidienst haben Sie?

[Eingabefeld] _____ Jahre (Angaben in 0,5 Schritten sind möglich)

1. Arbeiten Sie/haben Sie bei der Kriminalpolizei gearbeitet?

☐ Ja ☐ Nein
